# Identification of Pathogen Signatures in Prostate Cancer Using RNA-seq

**DOI:** 10.1371/journal.pone.0128955

**Published:** 2015-06-08

**Authors:** Yunqin Chen, Jia Wei

**Affiliations:** AstraZeneca, R&D Information, 199 Liangjing Road, Zhangjiang Hi-Tech Park, Shanghai, 201203, China; University of Ulster, UNITED KINGDOM

## Abstract

Infections of the prostate by bacteria, human papillomaviruses, polyomaviruses, xenotropic murine leukemia virus (MLV)-related gammaretroviruses, human cytomegaloviruses and other members of the herpesvirus family have been widely researched. However, many studies have yielded conflicting and controversial results. In this study, we systematically investigated the transcriptomes of human prostate samples for the unique genomic signatures of these pathogens using RNA-seq data from both western and Chinese patients. Human and nonhuman RNA-seq reads were mapped onto human and pathogen reference genomes respectively using alignment tools Bowtie and BLAT. Pathogen infections and integrations were analyzed in adherence with the standards from published studies. Among the nine pathogens (Propionibacterium acnes, HPV, HCMV, XMRV, BKV, JCV, SV40, EBV, and HBV) we analyzed, Propionibacterium acnes genes were detected in all prostate tumor samples and all adjacent samples, but not in prostate samples from healthy individuals. SV40, HCMV, EBV and low-risk HPVs transcripts were detected in one tumor sample and two adjacent samples from Chinese prostate cancer patients, but not in any samples of western prostate cancer patients; XMRV, BKV and JCV sequences were not identified in our work; HBV, as a negative control, was absent from any samples. Moreover, no pathogen integration was identified in our study. While further validation is required, our analysis provides evidence of Propionibacterium acnes infections in human prostate tumors. Noted differences in viral infections across ethnicity remain to be confirmed with other large prostate cancer data sets. The effects of bacterial and viral infections and their contributions to prostate cancer pathogenesis will require continuous research on associated pathogens.

## Introduction

As the second most common cause of cancer-related death among men [1], prostate cancer (PCa) remains a great public health concern. A significant amount of evidence has revealed that chronic inflammation can be associated with the onset of PCa [2, 3]. Chronic inflammatory infiltrates are common findings in prostate tissue samples and pathogen infections are considered to be one possible cause thereof.

It is known that Propionibacterium acnes (*P*. *acnes*) play an important role in human health and disease [4]. *P*. *acnes* in skin generally has a positive effect on human health by preventing the colonization of pathogenic microorganisms, but when the host becomes compromised (trauma, injury or alterations in immune status), it can display pathogenic potential [5, 6]. The presence of *P*. *acnes* has been strongly correlated with histological inflammation, suggesting that this bacterium might be linked to cancer development [7, 8]. Several studies have demonstrated the high prevalence of the Gram-positive bacterium *P*.*acnes* in the prostate tissues of men diagnosed with prostate disease [9, 10]. Infection of a prostate epithelial cell line with *P*. *acnes* induces a strong host inflammatory response and transformation, which could be a trigger for cancer initiation or progression [6, 10].

Viruses cause 10–15% of all human cancers. The association between infections caused by DNA viruses and the development of tumors is well established in many cancer types. Viruses associated with human cancers are known as 'tumor viruses'. Most of these viruses are capable of integrating into the host genome and immortalizing the target cell in order to facilitate their own replication. The infected cell expresses the viral genes, which are able to induce cell growth, proliferation and prevent apoptosis. For example, a high hepatitis B virus (HBV) load and chronic hepatitis B (CHB) infection increases the risk of developing hepatocellular carcinoma. HBV is a DNA virus that can integrate DNA into the host genome thereby increasing the yield of transactivator protein HBxAg. HBxAg is involved in many metabolic pathways [11]. Recently, some researchers have identified recurrent HBV integration events at the known and putative cancer-related genes such as TERT, MLL4 and CCNE1. These genes demonstrated upregulated gene expression in tumors but not in normal tissues [12]. The elucidation of tumor-DNA virus associations will enhance our fundamental knowledge of oncogenesis mechanisms and provide a foundation for cancer prevention initiatives.

More and more research has indicated that viral infections may lead to chronic or recurrent inflammation of the prostate and even initiate or promote carcinogenesis [13–15]. Viral products are able to interact with the interferon signalling pathway and induce cell transformation synergistically [16]. A number of viruses are reported to be associated with prostate cancer or prostate infections, namely Human papillomaviruses (HPVs), polyomaviruses (BK, JC, and SV40), and members of the herpesvirus family (HCMV, EBV) [17–21].

HPV is now recognized as one of the major causes of cervical cancer [22]. High-risk HPVs have also been frequently identified in both benign and malignant prostate tissues [23]. There are more than 100 different types of HPV and over 30 of these types are transmitted sexually, making HPV the most common sexually transmitted disease. The various HPV types are divided into two super categories- those that are more likely to develop into cancer and those that are less likely. The so-called "high-risk" forms are more likely to lead to the development of cancer, while "low-risk" viruses rarely develop into cancer.

HCMV infection typically goes unnoticed in healthy people, though it can be life-threatening for the immuno-compromised, such as HIV-infected persons, organ transplant recipients, or infants. After infection, HCMV has the ability to remain latent within the body over long periods. Eventually, it may cause mucoepidermoid carcinoma and possibly other malignancies. It is also reported that HCMV may be associated with prostate cancer [19, 24]. The Epstein-Barr virus (EBV), best known as the cause of infectious mononucleosis, is implicated in some malignant lymphomas and lymphoepithelioma-like carcinomas [25].

Polyomaviruses are small (40–50 nanometers in diameter) non-enveloped DNA viruses, and icosahedral in shape. They are potentially oncogenic (tumor-causing); and often persist as latent infections in a host without causing diseases, but may cause tumors in a host of a different species, or a host with an ineffective immune system. The polyomaviruses that infect humans, including the BK viruses (BKV), JC viruses (JCV), and simian virus 40 (SV40), typically cause infections that are subclinical and persistent [18].

The Xenotropic murine leukemia virus-related virus (XMRV) was first discovered in patients with PCa and later in patients with chronic fatigue syndrome (CFS) [26, 27]. Some further studies have provided evidence of XMRV infection in PCa [28–31] though its association with CFS and PCa has been largely discredited [32, 33]. Recent studies have suggested that the presence of XMRV may be a result of mouse DNA contamination [34, 35]. Because some results which indicate the presence of XMRV in PCa cannot be wholly attributed to sample contamination [36], here we examined the possible linkage between XMRV and PCa.

Uncovering the genomic effects of known pathogens in PCa remains a challenge. While most PCa research has focused on the PCR-based targeted detection of viruses, the advancement of next generation sequencing technology makes whole genome interrogation possible. Our goal is to analyze RNA-seq data from Chinese and western PCa patients in order to identify pathogens and their integration within the host genomes.

## Materials and Methods

### Three RNA-seq data sets

#### Data set 1: single-end miRNA-seq data of a pooled biopsy-proven PCa sample and a pooled control sample of Australian patients without detectable cancer

Small RNA sequencing was used to profile and compare miRNAs in the non-sperm cellular fraction of seminal fluid from men with biopsy-proven cancer (a pooled sample from 6 men) and men with elevated serum PSA but negative biopsy results (a pooled sample from 6 men) [37]. RNA was extracted using the Trizol reagent (Life Technologies) and cleaned up using RNeasy Mini kits (Qiagen). The single-end miRNA-seq data was downloaded from EBI ENA (http://www.ebi.ac.uk/ena/data/view/SRP041082).

#### Data set 2: paired-end mRNA-seq data of PCa tissues from Caucasian patients

Transcriptomes (polyA+) of 20 prostate tumors and 10 matched adjacent tissues were sequenced using the Illumina GAII platform. RNA was extracted from samples using the Ribopure kit (Ambion). Total RNA samples were processed for transcriptome sequencing using the Illumina mRNA-seq protocol. The pathological status of tumor samples was confirmed before processing, and the tumor samples had a tumor cell percentage >80% with Gleason scores ranging from 6 to 9 [38]. The mRNA-seq data was downloaded from EBI ENA (http://www.ebi.ac.uk/ena/data/view/SRX022060-SRX022089).

#### Data set 3: paired-end mRNA-seq data of Chinese PCa tissues

Prostate cancer and adjacent normal tissues from 14 patients obtained from the Shanghai Changhai Hospital were used as a cohort for RNA sequencing (using Illumina HiSeq 2000 sequencing machine). The tumor samples had Gleason scores ranging from 4 to 8 [39]. The mRNA-seq data was downloaded from EBI ENA (http://www.ebi.ac.uk/ena/data/view/ERP000550). Five samples (4T, 5T, 6N, 11T, 12N) had no available paired-end reads, which we analyzed using a single-end strategy.

All the data sets were generated in the FASTQ format. The sample collection, sequencing and clinical information of the 3 RNA-seq data sets was listed in S1 File. The average read length for Data set 1, 2, 3 is 49bp, 36bp, and 90bp respectively.

### Pathogens investigated in our work

We did a systemic literature review of viruses and bacteria reported in prostate cancer and finally selected seven viruses: HPV, HCMV, XMRV, BKV, JCV, SV40, EBV and one bacterium- Propionibacterium acnes (*P*. *acnes*) to investigate their pathogen associations with PCa. We also used the HBV virus, which can cause hepatitis B and rarely occurs in patients with PCa as a negative control virus to evaluate our sequencing analysis results.

### Reference Sequences used for mapping

The reference sequences consisted of human and pathogen sequences. Human genome and transcriptome sequences were downloaded from the NCBI website (NCBI build 37). Propionibacterium acnes and all the viral genome and transcriptome sequences were also taken from the NCBI build 37.

### Pathogen detection and analysis of pathogen integration sites

#### mRNA-seq data analysis pipeline for Data set 2 and Data set 3

We used the NGS QC Toolkit (v2.3) [40] to eliminate bad mRNA-seq reads. Two criteria were used to select good reads: cutOffQualScore = 20 and cutOffReadLen4HQ = 90, meaning that 90% of the bases of a qualified read must have quality scores > = 20. All subsequent analyses were based on clean mRNA-seq reads.

Both Bowtie [41] and the BLAST-like alignment tool-BLAT [42] were used in our virus detection pipeline. Bowtie is an ultrafast read aligner which can quickly map tens of millions of single-end or paired-end reads. We used Bowtie2 (version 2.1.0) and applied default parameters to carry out the alignment.

BLAT is an alignment tool like BLAST, but structured differently. It can efficiently map short reads across exon—exon junctions and identify novel splice junctions. Here we use standalone BLAT v.35. The parameters applied to align reads with reference sequences are as follows: minScore = 20, minIdentity = 88, stepSize = 5, and combined—fine and no—fine mode.

**Fig 1:**
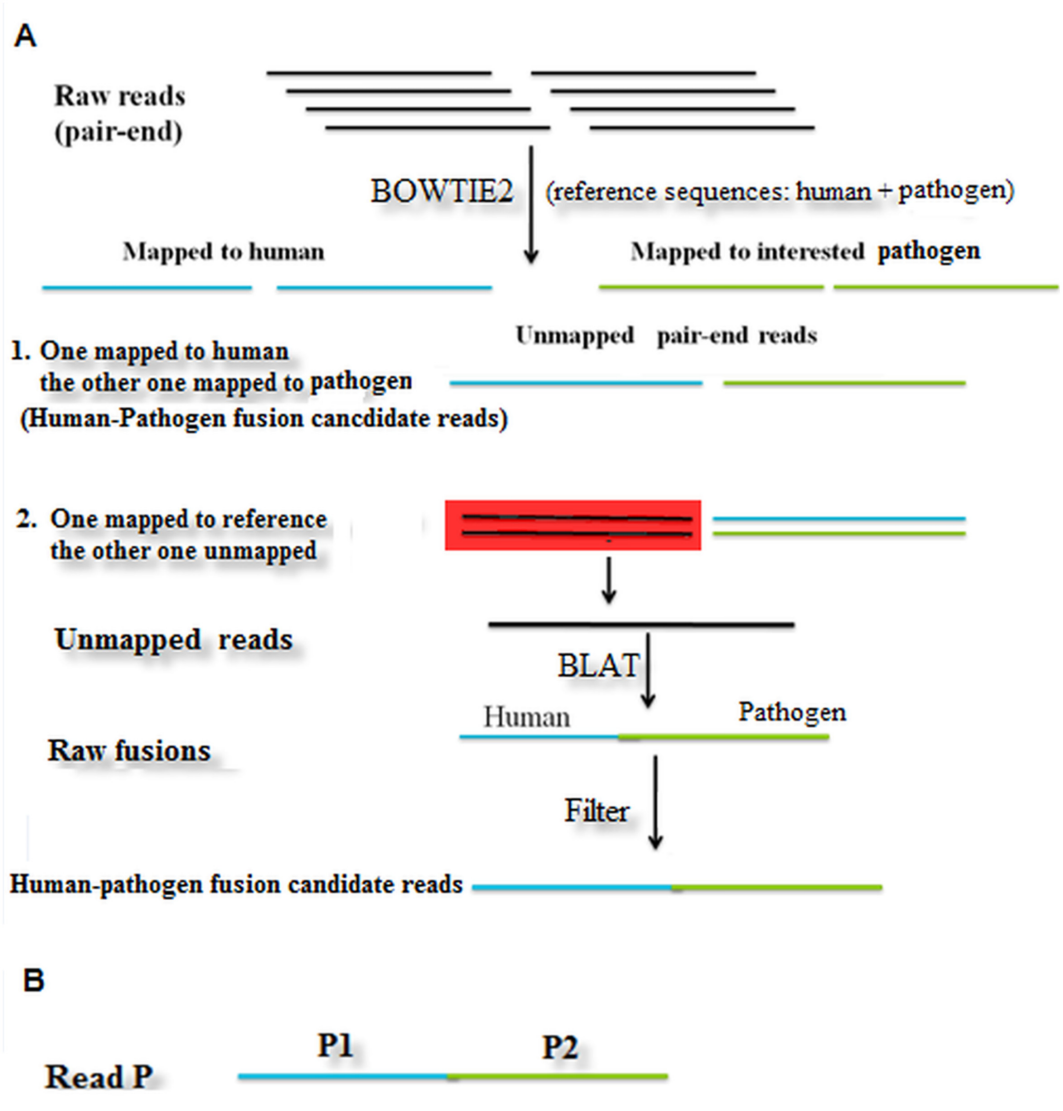
(A) Pathogen detection pipeline (B) Fusion Reads with one part (P1) mapped to human sequences and the other part (P2) mapped to pathogen sequences. Reads marked in blue are mapped to human sequences, reads marked in green are mapped to pathogen sequences, reads marked in black are unmapped.

Before mapping the reads, we performed an alignment between human and pathogen sequences to identify consensus sequences, which were used to filter false fusions. As shown in Fig 1A, raw reads were first mapped to human and pathogen reference sequences with Bowtie2, and then unmapped reads (only for length (read) >30) were picked out to perform local alignment with BLAT. If two paired-end reads were uniquely mapped with one read mapped to human sequences and the other read mapped to specific pathogen sequences, the paired-end reads were considered a human-pathogen fusion candidate read. If a read from one paired-end reads was uniquely mapped to human or pathogen sequences, and the other end read was uniquely from two parts: one part mapped to human sequences and the other part to pathogen sequences, it was labeled a raw human-pathogen fusion event. After detecting all the raw fusion events, we applied our criteria to filter out false positive reads and select fusion candidates.

For every single-end read with one part (P1) mapped to human sequences and the other part (P2) mapped to pathogen sequences (see Fig 1B), the following criteria were used for fusion filtering:

1. It is required that P1 is strictly mapped to human sequences and P2 is strictly mapped to pathogen sequences. Neither P1 nor P2 may overlap with consensus sequences.
2. The mapped ratio P1/(P1+P2) or P2/(P1+P1) < = 0.8.
3. P1 or P2 should be uniquely mapped.
4. P1 or P2 should not have low-complexity sequences.

After getting the fusion candidate reads, we mapped them to the human-pathogen consensus sequences to remove false positive fusions in cases where the fusion reads likely came from consensus sequences. Finally, we also manually checked their reliability by browsing their alignment using the web tool BLASTN (http://blast.ncbi.nlm.nih.gov/Blast.cgi) to ensure that the P1and P2 regions were mapped onto human and pathogen sequences.

#### MiRNA-seq data analysis pipeline for Data set 1

MiRNA-seq is a type of RNA-seq, which uses next-generation sequencing technology to sequence MicroRNAs. First, we filtered out the raw reads with a base quality score of less than 20. Then we removed the 3’ sequencing adapter using a BioPerl script (available at http://www.bioperl.org/wiki/Removing_sequencing_adapters). There were small RNAs like degraded mRNA fragments, rRNAs, tRNAs, miRNAs, siRNAs in the cleaned reads.

Because our purpose is to detect whether pathogens exist in the samples using the miRNA-seq data, we analyzed the clean reads using the mRNA-seq pipeline for single-end reads.

#### Measuring pathogen representation

In our study, quantified pathogen representation is determined by a measure of the overall count of mapped reads onto the pathogen genome and the expressed pathogen mRNAs in human samples.

We calculated the expressed pathogen mRNA levels by excluding non-transcribed pathogen genome elements. This eliminates and reduces the potential of nonsense reads and the number of non-transcribed pathogen genomic elements from the data pool. The transcript level and gene level counts were calculated and FPKM (fragments per kilobase of exon per million fragments mapped) were normalized using Cufflinks [43]. Then an FPKM filtering cutoff of 1.0 [44] or transcript count > = 2 was used to determine the level of expressed transcripts. Any pathogen expression level below the cutoff was labeled as having no obvious mRNA expression.

## Results

We analyzed three RNA-seq data sets of PCa samples- two from the western population and one from the Chinese population- using the methods outlined in the Materials and Methods section of this research paper. For data-set 1 of the miRNA seq data, 1% of approximately 100M raw reads were mapped to the human genome (because most of the reads should be mapped to the human miRNA library). For data-set 2, consisting of western prostate patients, the effective alignment count was about 17M on average. About 86% of reads were mapped to the human genome. For data-set 3, consisting of Chinese prostate patients, the effective alignment count was approximately 56M on average. About 85% of reads were mapped to the human genome.

### No virus but *P*. *acnes* detected in the PCa sample from Data set 1

None of the viruses were detected in either the pooled cancer sample or the normal sample from the western population, however *P*. *acnes* was identified in the cancer sample (see S2 File and Table 1). There were not high read loads of the *P*. *acnes* genome (about 77 reads mapped to each 100 kb on average) because of the limited number of small RNA sequences mapped to the reference sequences. The expressed genes with FPKM >1 were listed in Table 1. Intracellular protease / general stress protein 18 (YP_056816.1) responds to environmental stressors including extremes of temperature, exposure to toxins, and mechanical damage. Hypothetical protein PPA0381 (YP_055091.1) is likely to play a role in host-tissue biological degradation and inflammation [45]. Bacterial ABC transporters are essential in cell viability, virulence, and pathogenicity [46]. Fe(III) dicitrate ABC transporter (YP_056465.1) is participating in Iron ABC uptake systems which are important effectors of virulence.

**Table 1:** *P*. *acnes* genes expressed in Data set 1.

Gene id	Protein id	Protein name	FPKM for cancer sample (n = 6)	FPKM for control sample (n = 6)
gene137	YP_054855.1	reductase, ferredoxin	5.771	0
gene258	YP_054973.1	inorganic pyrophosphatase	12.5813	0
gene377	YP_055091.1	hypothetical protein PPA0381	18.245	0
gene558	YP_055264.1	hypothetical protein PPA2401	15.9918	0
gene1198	YP_055893.1	hypothetical protein PPA1186	8.3743	0
gene1397	YP_056085.1	imidazole glycerol phosphate synthase subunit HisF	10.5343	0
gene1795	YP_056465.1	Fe(III) dicitrate ABC transporter, ATP-binding protein	31.7288	0
gene2158	YP_056816.1	intracellular protease / general stress protein 18	23.8085	0
gene2186	YP_056844.1	hypothetical protein PPA2180	12.3472	0

Since the read length was < 60bp, we cannot use a single-end strategy to find human-bacteria fusions in these samples.

### No virus but *P*. *acnes* detected in both PCa and adjacent benign samples in Data set 2

There were no obvious virus-mapped reads detected in the 20 PCa samples nor in the 10 matched adjacent samples from western patients. However *the P*. *acnes* signature was detected in both cancer samples and adjacent samples (see S2 File). 14 out of 20 cancer samples and 9 out 10 adjacent benign samples had at least 2 expressed *P*. *acnes* transcripts.

Using 10 paired cancer and adjacent samples, we identified 7 cancer-specific genes from *P*. *acnes*, all of which were expressed in at least 3 out 10 cancer samples (see Table 2). Among them, bacterial topoisomerase I (YP_054960.1) is required for preventing the hypernegative supercoiling of DNA during transcription and plays an important role in the transcription of stress genes during the bacterial stress response [47]. And elongation factor G (YP_056556.1) promotes the translocation step in bacterial protein synthesis.

**Table 2:** Cancer-specific *P*. *acnes* genes in Data set 2: Western prostate cancer samples compared with matched adjacent samples.

Gene id	Protein id	Protein name	# Cancer sample
gene114	YP_054834.1	potassium-transporting ATPase subunit B	3
gene243	YP_054960.1	DNA topoisomerase I	4
gene460	YP_055173.1	dehydrogenase, myo-inositol 2-dehydrogenase	4
gene812	YP_055517.1	excinuclease ABC subunit B	3
gene1052	YP_055753.1	major facilitator superfamily permease	5
gene1592	YP_056275.1	hypothetical protein PPA1574	3
gene1887	YP_056556.1	elongation factor G	3

Only expressed transcripts with an FPKM > = 1.0 in at least 3 out 10 cancer samples are shown.

We tried to find human-bacteria fusion in *P*. *acnes* identified samples, but no such paired-end reads where one read mapped to human sequences and the other mapped to *P*. *acnes* sequences were identified.

### Viruses and *P*. *acnes* identified in cancer and adjacent samples in Data set 3

In this study, there were 9 matched cancer and adjacent samples, 3 unpaired cancer samples and 2 unpaired adjacent samples available in Data set 3 (see Materials and Methods).

The reads from *P*. *acnes* were identified in all Chinese cancer samples and adjacent normal samples. Sample 7N, 7T, 8N had a high RNA level of *P*. *acnes* (see S2 File). All but 2 cancer samples have evidence of expressed *P*. *acnes* transcripts. However, we did not find cancer-specific *P*. *acnes* transcripts which were expressed in at least 3 cancer samples as our standard threshold.

Interestingly, some virus reads were detected in 3 Chinese prostate samples-cancer sample 7T and adjacent samples 7N, 8N- all from patients with stage III PCa and with high PSA scores (7T&7N: PSA 30.33; 8N: PSA10.4) (see S1 File).

All 3 samples were identified as having viral DNA loads, expressed transcripts and more than one kind of virus was detected in each. The infected virus profiles were similar across the 3 samples (see Table 3). The viruses with more reads were SV40, HCMV, EBV and low-risk HPVs (like HPV49, 50, 88,108) (see Table 3 and S3 File).

**Table 3:** Viral transcripts detected in Chinese prostate samples.

Virus genome	Virus name	Expressed gene #	7N. read counts	7T. read counts	8N. read counts
NC_009334.1	EBV type 2	39	367	902	592
NC_006273.2	HCMV	38	2079	1916	2795
NC_007605.1	EBV	30	3224	2735	3637
NC_001669.1	SV40	10	1450	916	1470
NC_001591.1	HPV49	4	39	65	70
NC_001595.1	HPV7	4	15	51	17
NC_008188.1	HPV103	4	26	11	45
NC_010329.1	HPV88	4	177	79	151
NC_001691.1	HPV50	3	176	127	171
NC_004104.1	HPV90	3	3	11	6
NC_004500.1	HPV92	2	21242	21970	14097
NC_005134.2	HPV96	3	17	19	34
NC_008189.1	HPV101	3	6	6	11

Expressed gene # column shows the number of expressed genes in each virus detected sample from RNA-seq data.

7N. read counts column shows the number of paired-end reads mapped to each viral reference sequence for sample 7N.

7T. read counts column shows the number of paired-end reads mapped to each viral reference sequence for sample 7T.

8N. read counts column shows the number of paired-end reads mapped to each viral reference sequence for sample 8N.

Similarly, we used the fusion detection pipeline to identify human-pathogen fusion events. We filtered the raw fusions using consensus sequences between each pathogen and human sequence. No viral or bacterial fusions were identified in Data set 3.

It is known that different *P*. *acnes* strains are associated with the prostate than those commonly found on the skin [48]. The draft genome sequences of two strains of *P*. *acnes* isolated from radical prostatectomy specimens (*P*. *acnes* P6 and *P*. *acnes* PA2) were reported [49]. We tried to use the genome sequences of 3 strains: KPA171202 (from skin), P6 and PA2 (from prostate), but the read counts were not high enough to detect the strain specific sequences of genes such as the housekeeping gene (RecA) or the putative hemolysin gene (Tly) in order to investigate the phylogenetic relationship between bacterial organisms [50, 51]. 16S rRNA is the gold standard for phylogenetic analysis. However, 16S rRNA could be removed during sequencing library preparation according to the protocols of RNA-seq technology. We did not identify any reads mapped to 16S rRNA.

## Discussion

Microorganism infections of the prostate by *P*. *acnes*, HPV, HCMV, JCV and BKV and. the recently discovered, XMRV have been suggested as important risk factors in the development of PCa [17, 19, 23, 29, 52, 53]. Until now, studies aimed at replicating the associations between these pathogen infections with PCa have yielded conflicting results across different ethnic groups. Our current study analyzed associations between pathogen infections among Chinese and western PCa patients using RNA-seq. To our knowledge, this is the first analysis of the role of these viruses and bacteria in the development of prostate tumors of both western and Chinese patients using NGS data.

The bacterium *P*. *acnes* was detected in all three data sets, in both cancer tissues and adjacent benign prostate tissues except in normal samples from healthy individuals. Due to data limitations, we cannot rule out the possibility that the *P*. *acnes* we discovered in the prostate tissue may have come from the skin. Our observation was consistent with previous reports that *P*. *acnes* were prevalent in both benign and malignant prostate tissues [9, 54]. It is highly likely that *P*. *acnes* were present in prostate cancer but not normal samples. *P*.*acnes* is believed to induce and maintain an inflammatory response by producing chemotactic factors that attract neutrophils [55] and the ability of *P*. *acnes* to survive phagocytosis in vitro has been confirmed [56]. Moreover, *P*. *acnes* releases proteases and other enzymes that contribute to tissue injury [45]. The host response to *P*. *acnes* is characterized by the production of proinflammatory cytokines such as tumour necrosis factor-α (TNF-α), interleukin 1-α (IL-1α) and interleukin 8 (IL-8) [57]. *P*.*acnes* has been shown to induce these cytokines in both macrophages and keratinocytes through the TLR2 and TLR4 signaling pathways [58, 59]. *P*. *acnes* may pose an increased risk for PCa given its role in inflammatory processes.

Another interesting finding in our study is that we observed the difference between adjacent tissues surrounding tumors and normal tissues from healthy individuals. The *P*. *acnes* did not exist in normal samples from healthy individuals but appeared in all adjacent samples of PCa patients in our work. The tissues adjacent to prostate tumors may also be undergoing tumor related changes, therefore careful characterization of these different tissues is necessary to understand the molecular changes leading up to PCa [60].

No viruses were detected in western prostate samples in Data set 1 and 2. But viruses like HCMV, EBV, SV40 and low-risk HPVs were identified in some of the Chinese prostate tissues (both cancer and adjacent samples) in Data set 3. The presence of both HPV and EBV gene sequences were present in an equal proportion of normal, benign, and PCa specimens from Australian males. However, using a range of analytical techniques including the in situ polymerase chain reaction (IS-PCR) and the standard liquid PCR before finally sequencing the product [21], no DNA virus transcripts were detected in the PCa samples using RNA-seq data from the TCGA Portal (https://tcga-data.nci.nih.gov/tcga/) [61, 62]. The different results from different studies are most likely attributable to sample differences: ethnic difference, pathological difference, etc. The Chinese sample 7N, 7T, 8N were infected by both *P*. *acnes* and other viruses. The presence of multiple viral sequences in the same prostate tumor sample is particularly noteworthy because these observations confirm the assessment of Zambrano et al.[63] that the prostate is a habitat for multiple viral and other infections, some of which may have oncogenic potential. Because only 2 out of 14 patients (patient No.7 and patient No.8) in Data set 3 had viral infections, no solid conclusion can be drawn as to whether those viruses contribute to the development of PCa.

XMRV and other viruses were not detected in any of the three datasets. Our results are consistent with the majority of published studies on XMRV, which show that XMRV is not present in humans [33, 64–66]. The absence of high risk HPV16 and HPV18 from any of the data sets analyzed indicates that high risk HPV is not associated with PCa in the investigated patients.

It has been reported that viral and bacterial integration occurs in the human somatic genome and may play a role in carcinogenesis. The fact that we did not detect any human-bacteria or human-virus fusion in any data set may suggest that *P*. *acnes* and the 4 viruses (HCMV, EBV, SV40, low- risk HPVs) may not pose severe risks for the development of prostate cancer.

Our study yielded a number of interesting findings pertaining to the pathogen signatures of PCa patients. The major limitations of this study are the small RNA-seq data set size and the lack of available tissue samples for validation. While the findings of this study, especially the presence of viruses in Chinese PCa patients, remain to be validated by large prostate data sets, our analysis sheds light on the emerging application of RNA-seq technology in detecting pathogen signatures in human cancers and elucidating novel mechanisms of pathogen-driven cancer pathogenesis.

## Supporting Information

S1 File Prostate sample information for the three data sets. (XLSX)

S2 File Mapped read coverage of *P*. *acnes* sequences in the three data sets. (XLSX)

S3 File Viral genes expressed in samples 7N, 7T, 8N of Data set 3. (XLSX)
